# Development of highly inhomogeneous temperature profile within electrically heated alkali silicate glasses

**DOI:** 10.1038/s41598-019-39431-8

**Published:** 2019-02-26

**Authors:** Charles T. McLaren, Craig Kopatz, Nicholas J. Smith, Himanshu Jain

**Affiliations:** 1 Department of Materials Science and Engineering, Lehigh University, Bethlehem, PA 18015 USA; 2 Corning Incorporated, Corning, NY 14830 USA

## Abstract

According to Joule’s well-known first law, application of electric field across a homogeneous solid should produce heat uniformly in proportion to the square of electrical current. Here we report strong departure from this expectation for common, homogeneous ionic solids such as alkali silicate glasses when subjected even to moderate fields (~100 V/cm). Unlike electronically conducting metals and semiconductors, with time the heating of ionically conducting glass becomes extremely inhomogeneous with the formation of a nanoscale alkali-depletion region, such that the glass melts near the anode, even evaporates, while remaining solid elsewhere. *In situ* infrared imaging shows and finite element analysis confirms localized temperatures more than thousand degrees above the remaining sample depending on whether the field is DC or AC. These observations unravel the origin of recently discovered electric field induced softening of glass. The observed highly inhomogeneous temperature profile point to the challenges for the application of Joule’s law to the electrical performance of glassy thin films, nanoscale devices, and similarly-scaled phenomena.

## Introduction

Joule’s first law, sometimes also referred as the Joule–Lenz law, describes heat produced from passing of electrical current through a conductor (resistor)^1,2^. Most simply, it states that heat is produced in proportion to the square of electrical current that passes through a material. This statement is readily verified with homogeneous conductors or semiconductors. For inhomogeneous materials, such as composites, the law can be suitably extended by restating that heat is produced in proportion to the square of *local* electrical field as constant current passes through the sample^3,4^. Thus, if the material comprises of regions of varying resistance, there will be corresponding variation of field according to Ohm’s law, and hence the heat produced. Such trivial expressions of Joule heating are well-established^5,6^. However, much less is known about the manifestation of Joule’s law for the case of a material that is homogeneous to begin with, but wherein the effect itself (by migrating charge-carriers) may modify the material over time (of course, the validity of fundamental Joule effect at the local level remains unquestioned, at least for the time and length scales investigated here and encountered in common applications)^7^. A scenario of such a time dependent, electric field-induced modification of a material was proposed to explain a recently discovered phenomenon: the electric field induced softening (EFIS) of model alkali silicate glasses. EFIS, as described below, has been the primary motivation behind this study, but its implications are far-reaching for the electrical performance of ionic solids such as common glasses and crystalline dielectrics, and even more so in thin film and nanoscale devices where inhomogeneity can become deleterious more readily.

Glasses have been used in a variety of electrical applications, most notably as insulators, substrates for microelectronics, discrete dielectric elements, etc^1,8^. They serve multiple purposes within electrical components, which include keeping electrical contacts from shorting by allowing little to no current flow, increasing the effective capacitance of a component, and/or allowing for high voltage operation of a component without dielectric breakdown. A failure of the last condition occurs when the local electric field strength exceeds the material’s ability to resist current flow^1^. Once a critical electric field (dielectric strength) has been exceeded, breakdown of the material occurs and allows for large current flow^9^. This process has been observed in many glasses, thin insulating films, and other dielectrics, but is not always easily measured^10,11^. Factors influencing dielectric breakdown strength often link to intrinsic material properties, but also extrinsic factors such as voltage rise rate, surface defects, and external temperature. For example, as the temperature of silicate glass increases, its dielectric strength decreases dramatically^9^. A common mechanism associated with typical field conditions in various glasses and polymers is the so-called thermal runaway due to high power densities and Joule heating leading to a catastrophic rise in current flow^11^. Thermal runaway can soften, melt and ultimately vaporize the dielectric material if not limited. Modeling of various kinds has estimated temperatures to rise to the order of 1000’s of degrees Celsius^1,10,12^. However, little direct experimental data is available on temperature during the dielectric breakdown process in glass until recently under DC-EFIS conditions^13^.

Modern technologies and processing techniques have been able to take advantage of the dielectric properties of glass^14–18^. For example, glass has been processed in a variety of innovative ways to create waveguides, enable through-glass vias for use as dielectric components, or even create surface structures for use with chip-on-glass applications, integrated circuits, etc^14–16^. Glass softening via EFIS at much lower furnace temperatures (T_furnace_) than that obtainable through conventional heating has been demonstrated using both externally applied AC and DC^19–21^. Flash sintering of glass powder is believed to be intimately related to EFIS^22,23^. This phenomenon has been exploited in improving the fabrication of quantum dots in a glass^24^. The mechanism of EFIS assumes multiple cascading events under applied voltage at elevated temperatures^20^. The process has largely been attributed to, first, the formation of a highly resistive alkali ion depletion layer near the anode which undergoes dielectric breakdown^20^. It is then followed by large current flow and thermal runaway through the sample, eventually resulting in viscous flow of the glass samples. Intriguingly, wisps of vapor could be visibly seen near the glass-anode interface during resistive heating and thermal runaway in EFIS (see Supplemental Video in)^19^. This observation is an indication that highly non-uniform, immense heating occurs, which could not be predicted from classical Joule’s law proposed for homogeneous bulk materials. The present study aims to assess the applicability of Joule’s law on the micrometer scale to electrical heating of ionic solids, especially common silicate glasses, as well as the mechanism of EFIS and dielectric breakdown of glass by measuring accurately the sample surface temperature using *in situ* thermal infrared imaging with state-of-the-art infrared thermal imaging^25^. The initiation temperature and alkali ion depletion layer thickness (δ) dependencies for thermal runaway are calculated using finite element analysis (FEA), which provide insight into the effect of variables that are hard to control experimentally.

## Results

Manifestations of localized Joule heating are most clearly seen as physical softening and large deformation of glass samples, which should be otherwise solid at the ambient temperature of the furnace wherein the EFIS experiment is performed. Therefore, we summarize the key observations of EFIS first, which are similar to those reported previously: When heating the sample at a constant rate and applied voltage, thermally stimulated polarization current exhibited a large step-change at some temperature wherein 30 seconds or less, the glass softens and deforms under the applied load^19–21^. As typical examples of the electrical current and power through glass samples, experimental data are shown in Supplementary Fig. 1 for 5L5NS glass during the EFIS event with 200 V DC applied. The voltage drop across the samples is seen to decrease as EFIS occurs, which in turn slightly reduces power dissipation.

**Figure 1:**
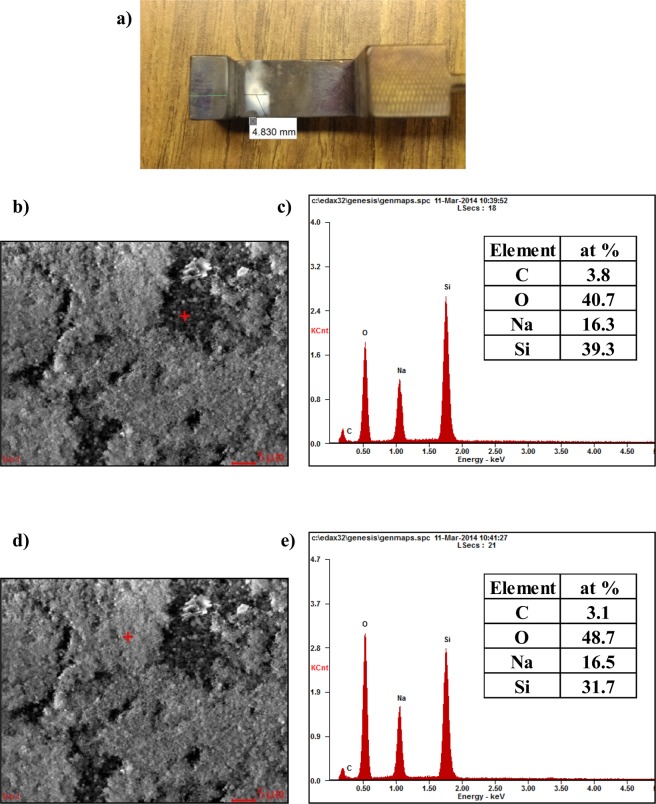
After testing 2L8NS with 200 V/cm (**a**) buildup of white powder was observed on the compression hook near the anode. The powder was investigated using EDS where (**b**) spot 1 is indicated by the red crosshairs with its (**c**) collected spectrum and (**d**) spot 2 again indicated by the red crosshairs with its (**e**) collected spectrum. The semi-quantitative compositions are given for each spot in atomic percent.

During and following this EFIS effect under 200 V DC, a white powder buildup was noticed on the loading structure adjacent to the glass sample, as shown in Fig. 1 for a lithium sodium silicate glass (2L8NS, see Table 1 for exact composition). The EDS spectra for this powder indicate the presence of carbon, oxygen, sodium and silicon. Lithium was not detectable due to x-ray absorption by the detector window. No elements besides those present in the glass and carbon paste electrodes were detected. The close similarity of the powder composition to the 2L8NS glass indicates that the powdery material was vaporized glass that had condensed on the tooling surfaces, in all likelihood after immense heating.

**Table 1:** Glass compositions and their respective glass transition temperatures (T_g_) measured by DSC at 10 °C/min.

Glass Type	Composition	T_g_ [°C]
NS	0.33 Na_2_O • 0.67 SiO_2_	469.7 ± 0.9
2L8NS	0.33 [0.2 Li_2_O • 0.8 Na_2_O] • 0.67 SiO_2_	424.3 ± 0.7
5L5NS	0.33 [0.5 Li_2_O • 0.5 Na_2_O] • 0.67 SiO_2_	420.4 ± 0.3

**Figure 2:**
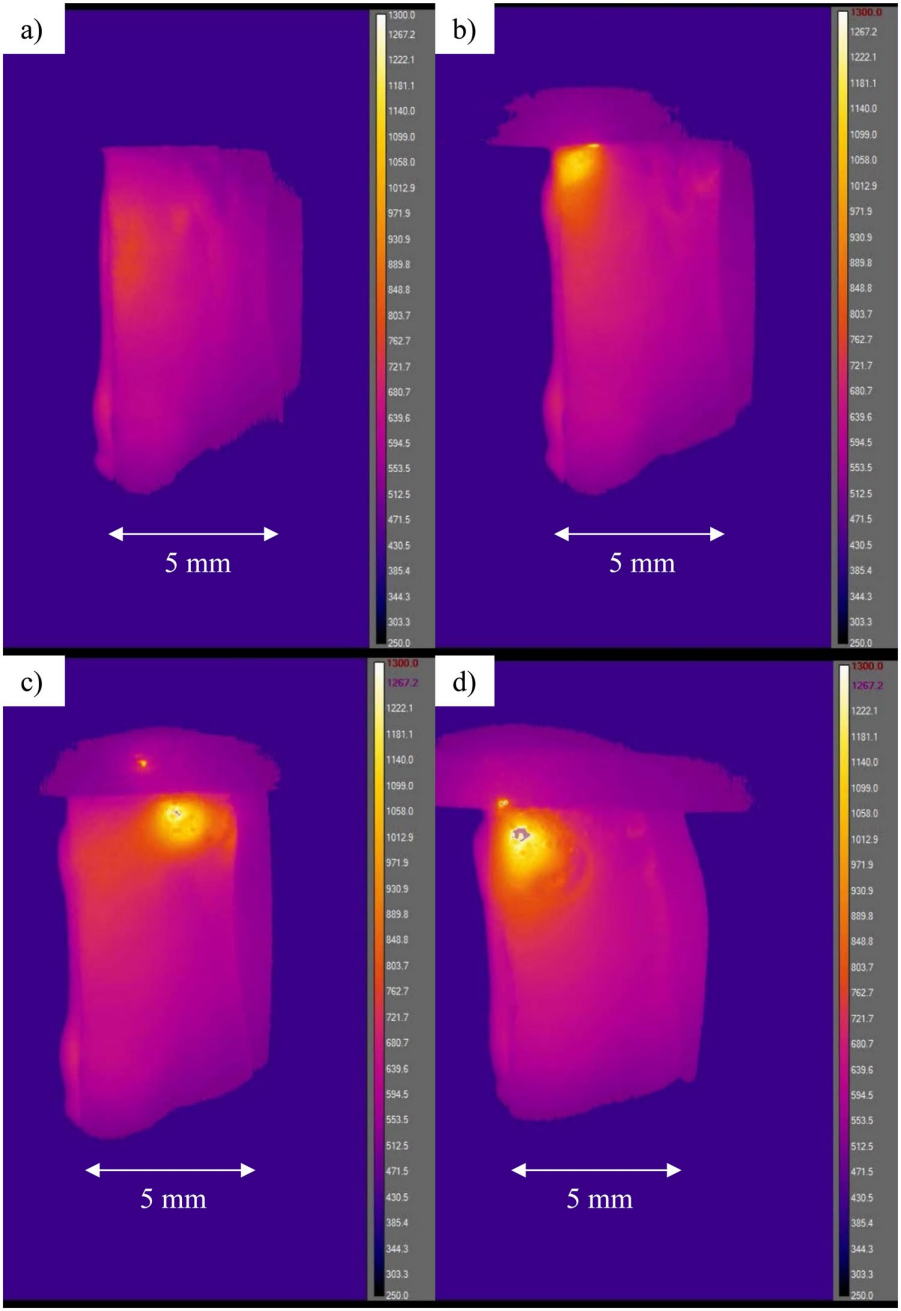
Series of infrared images of NS with 200 V applied at T_furnace_ = 320 °C, captured from video at (**a**) start of thermal runaway (at 41 s), (**b**) localized heating during EFIS (at 43 s), (**c**) anode discharge following intense heating (at 45 s) and (**d**) localized glass softening (at 54 s). Note: Anode is located at top of sample and temperature scale ranges from 250 °C up to 1300 °C.

**Figure 3:**
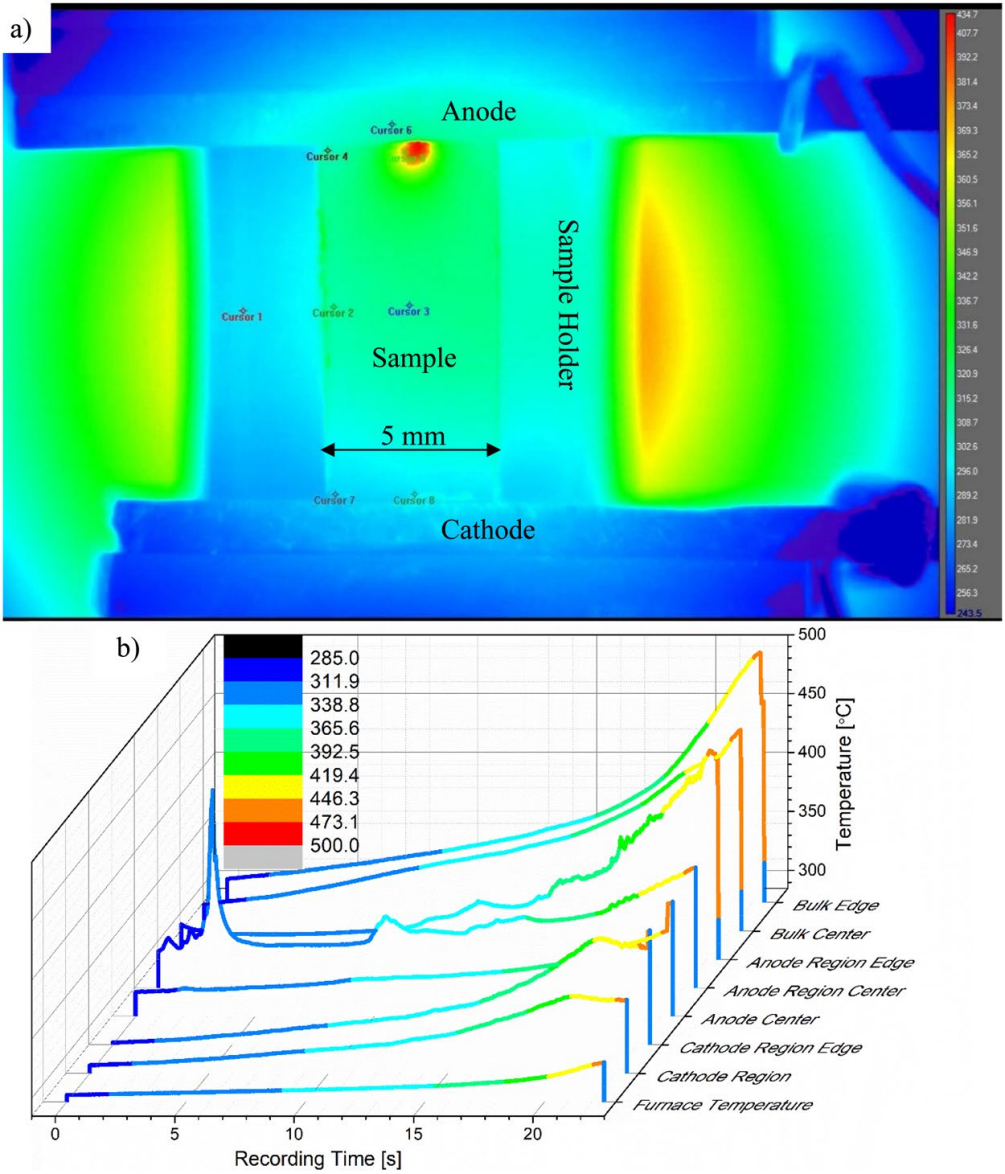
IR image of NS with 200 V DC at T_furnace_ = 305 °C for (**a**) temperature cursor locations at 4 s and (**b**) cursors temperature average of 3 × 3 pixels for anode, bulk and cathode glass regions along with furnace and anode temperatures for the first 22 s of EFIS thermal runaway.

The temperature of several samples of each glass composition listed in Table 1 was measured *in situ* under EFIS conditions with applied DC voltage ranging from 100 V to 200 V at T_furnace_ varying from 25 °C to 450 °C. A series of representative IR images during the thermal runaway process shows NS glass at 200 V and T_furnace_ = 320 °C (see Fig. 2). (A video of the thermal runaway process for 5L5NS with 200 V applied can be found in Supplemental Material). All images were captured within 13 seconds by video and reported with their associated temperature on the right side of the frame. They show four stages of glass heating under the application of electric field: At first, the glass begins to resistively heat locally near the anode much more than anywhere else, as seen in Fig. 2a. Thermal imaging of the same NS sample with 200 V DC using a relative-difference color palette visibly shows a non-uniform localized heating behavior at the onset of thermal runaway (see Fig. 3a). The corresponding temperature profiles at various locations on the sample are displayed with respect to time in Fig. 3b, highlighting the range of temperatures observed and their evolution with time.

Returning to Fig. 2b, next the positive feedback of thermal runaway builds up, quickly causing intense heating of the glass near the anode to above 1,300 °C—the maximum detectable temperature of the IR camera used. The temperature change was clearly non-uniform, manifesting in a relatively localized “hot spot” that moved laterally around the interface in seemingly random directions. In previous reports^19,20,26^, small, localized areas of intense photoemission were similarly observed in optical images, and noted to also “wander” around the anode-interface region of the glass, as seen in Supplemental Video^19,26^. The same movement mimicked by the intense local heating of the glass is believed to be correlated to the motion of photoemissions. Sparks near the anode and sometimes within the graphite anode are then observed, as shown in Fig. 2c. Lastly, in Fig. 2d, the glass begins to soften locally and the generated heat diffuses into the bulk sample by thermal conduction, thereby extending softening/deformation to the rest of the sample.

An interesting feature was observed occasionally during some EFIS experiments, such as on 2L8NS glass with 150 V DC applied. IR video shows the growth of a dendrite-shaped, visibly brown phase within the glass from cathode toward the anode during dielectric breakdown of the glass. This is highlighted by yellow circles in Supplementary Fig. 2. It is consistent with EDS measurements of a sodium-rich phase near the cathode and has been noted by others^27–30^.

**Figure 4:**
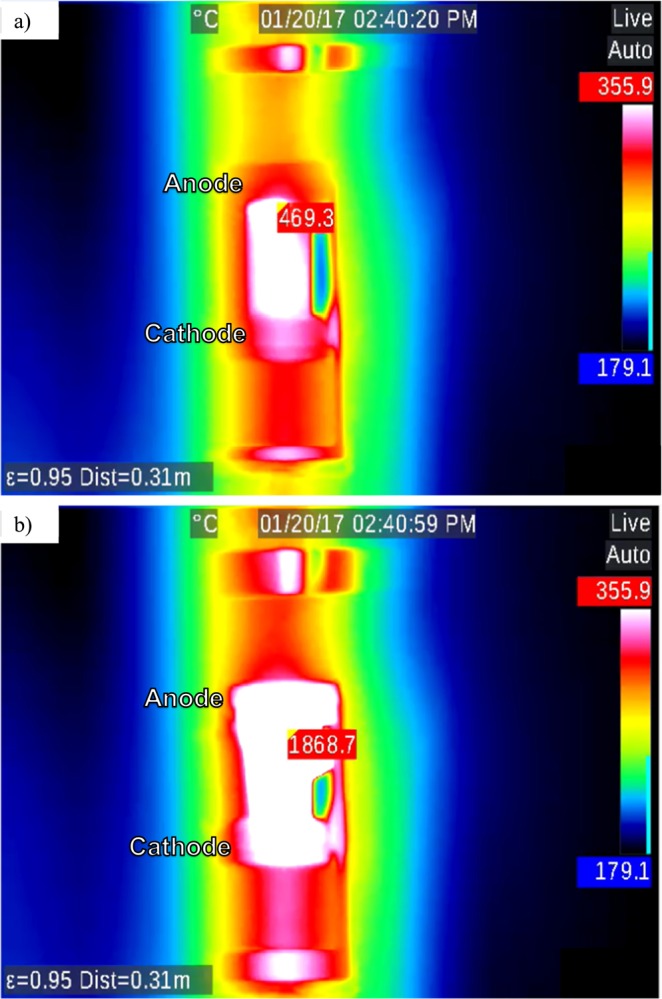
IR images of NS with 150 V applied in DC at (**a**) run time = 2069.4 s and T_furnace_ = 353.5 °C and (**b**) run time = 2108.5 s and T_furnace_ = 363.6 °C. Note: Color scale was not updated but hottest pixel is identified with surface temperature reading.

**Figure 5:**
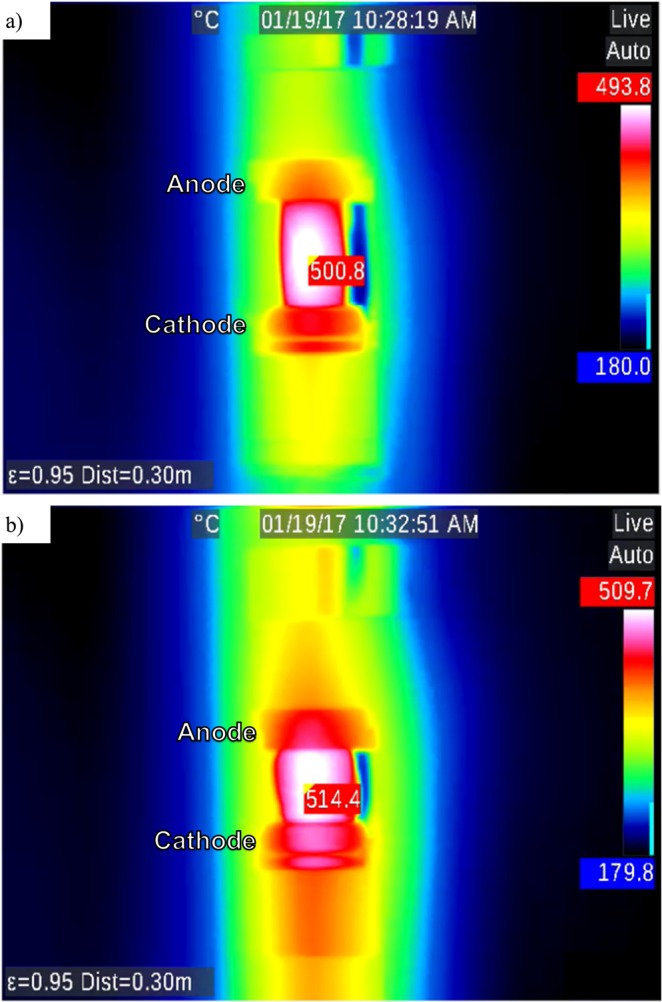
IR images of NS with 150 V 1000 Hz applied in AC at (**a**) run time = 2130 s and T_furnace_ = 385.3 °C and (**b**) run time = 2403 s and T_furnace_ = 428.6 °C. Note: Color scale was not updated but hottest pixel is identified with surface temperature reading.

In order to measure temperatures higher than 1300 °C, a second thermal camera was used with two NS samples. First, 150 V DC was applied across a sample and heated at a rate of 10 °C/min. Two thermal images of this sample are shown in Fig. 4a, which were recorded at time, t = 2069.4 s since the beginning of EFIS experiment corresponding to a T_furnace_ of 353.5 °C, and in Fig. 4b at t = 2108.5 s with a T_furnace_ of 363.6 °C. The color scale is the same for the two images of Fig. 4. Here, the hottest pixels in the two images were identified to have local temperatures of 469.3 °C and 1868.7 °C, as indicated in Fig. 4a,b, respectively. Thus, the local temperature increased by ~1400 °C in less than 30 s, while the T_furnace_ increased only by 10.1 °C! Across the second NS sample, 150 V AC of 1 kHz frequency was applied. Two thermal images of this sample are shown: Fig. 5a, at t = 2130 s corresponding to T_furnace_ 385.3 °C and the highest local sample temperature of 500.8 °C, and Fig. 5b, at t = 2403 s with T_furnace_ = 428.6 °C and the highest local sample temperature of 514.4 °C.

**Figure 6:**
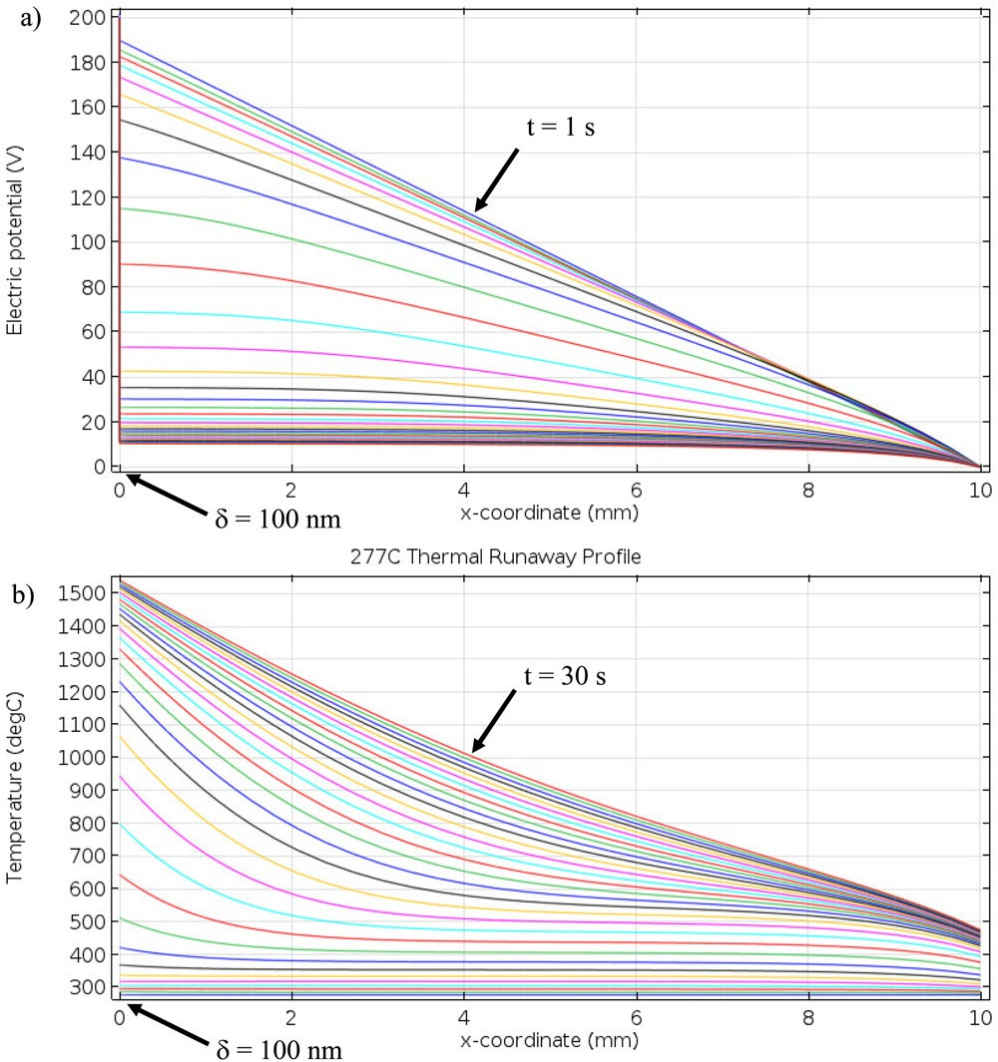
Finite element analysis of (**a**) electric potential (increasing time trending toward bottom left) and (**b**) temperature profiles (increasing time trending toward top right) of NS resulting from resistive heating at 1 s intervals over 30 s total with 200 V DC applied at an initial temperature of 277 °C and a fixed δ at 100 nm. The anode/glass interface is located at 0 mm while glass/cathode interface at 10 mm in the model.

Finite element calculations of thermal runaway, as caused by resistive heating of an ionic conductor, are shown in Fig. 6 for NS with 200 V DC applied. The electric potential is shown in Fig. 6a, whereas Fig. 6b overlays the calculated temperature profiles for time steps from 1–30 s in 1 s intervals. Here, the profile extends from anode (located at 0 mm) to cathode (located at 10 mm). During thermal runaway, the alkali ion depletion layer (fixed at 100 nm thickness) next to the anode begins to heat rapidly compared to the bulk of the glass due to its higher resistivity. After 30 s of runaway at a starting temperature of 277 °C (500 K), the temperature of the depletion layer increases to nearly 1525 °C. The decrease of temperature from anode to cathode is gradual, indicating that the sample heats up over a much thicker region (~ a few mm) than the width of depletion region (~100 nm) where power dissipation in the bulk can contribute.

**Figure 7:**
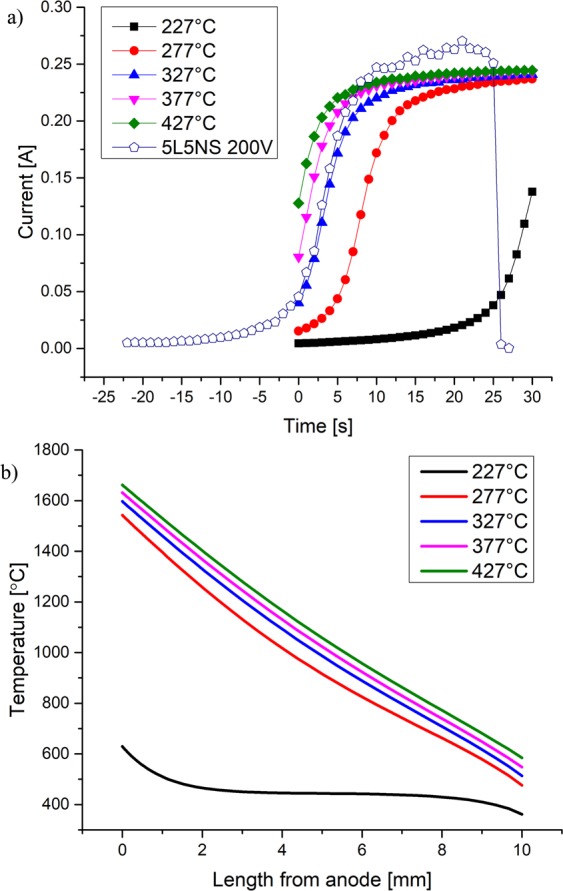
Model calculations of thermal runaway of NS at various initial temperatures with a δ of 100 nm for (**a**) current during 30 s of self-heating (solid symbols), and (**b**) thermal profile at 30 s. As an example of comparison with experiments, the open symbols in (**a**) are observed data for 5L5NS glass heated at a constant rate of 10 K/min under 200 V with EFIS occurring at 325 °C. Note: Current limit reached is a result of heat generation versus heat loss.

The effect of initiation temperature on thermal runaway by resistive heating was calculated using starting temperatures ranging from 227 °C (500 K) to 427 °C (700 K) in 50 °C intervals with a fixed value of depletion layer thickness, δ = 100 nm in Fig. 7. The profile in Fig. 6b is representative of the various initial temperatures of Fig. 7a. The main source of resistive heating is the current through the relatively ‘thin’ depletion layer, where the electric potential drop is large on the left side of Fig. 6a compared to rest of the bulk. The simulated current for each initial temperature is shown in Fig. 7a as a function of time to 30 s. At 227 °C, the current increases slowly at short times, and then rapidly at longer times — a behavior typical of a positive feedback system. Within 30 s, the temperature in the depletion layer is about 650 °C, which is about 425 °C above the initial temperature of 227 °C. For the higher initial temperatures of 277 °C to 427 °C the increase of current is a much faster exponential rise, as seen in Fig. 7a. Eventually, the current reaches an asymptote prior to the artificial current limiting boundary condition imposed on the system. Experimentally measured current for 5L5NS glass sample during EFIS with 200 V applied is also included in Fig. 7a (open symbols) to validate the theoretical model and assess its usefulness. In this regard, we note that the time scale and magnitude of the measured EFIS data agree with the theoretically modeled values reasonably well, suggesting that thermal runaway is an important, if not the only, factor in producing EFIS. As the initial starting temperature is increased, the highest temperature reached after 30 seconds also increased as shown in Fig. 7b. For example, at 277 °C, 327 °C, 377 °C and 427 °C the highest temperature calculated in the depletion layer was approximately 1575 °C, 1600 °C, 1625 °C and 1650 °C, respectively.

**Figure 8:**
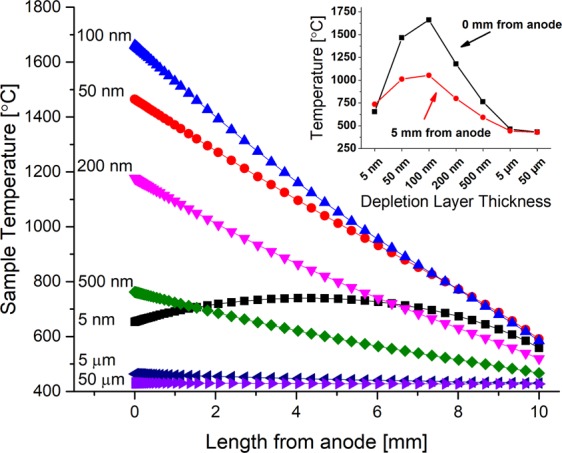
Thermal profiling at 30 s of self-heating as a function of depletion layer thickness, δ. Below 100 nm, heat loss from the depletion layer occurs rapidly. Above 100 nm the depletion layer resistivity limits the current as a negative feedback. Note: the inset shows the temperature relation 0 mm (black, squares) and 5 mm (red, circles) from the anode for each δ.

The dependence of the temperature profile on δ was also investigated with FEA. In this analysis, a pre-existing depletion layer was included in the model, and its thickness was varied from 5 nm to 50 µm. Initial temperature was held constant at 427 °C. For each value, the temperature profile across the sample after 30 s of self-heating is shown in Fig. 8 as a function of distance from the anode. At 5 nm thickness, the depletion layer resistance was low, preventing it from having much influence over the resistance of the bulk, therefore, the bulk of sample heated quicker than the depletion layer. This resulted in a thermal profile that was symmetric, with the center of the bulk being the hottest at about 750 °C. An increase of δ to 50 nm increased the resistance of the depletion layer, allowing for dramatic localized heating up to 1450 °C. The maximum temperature of the depletion layer was reached with 100 nm at 1650 °C. With δ > 100 nm, the sample did not experience as severe of a thermal runaway near the anode. It was calculated that as δ increased, the maximum current decreased, resulting in the suppression of thermal runaway (see Fig. 8). For δ = 50 μm, the sample temperature remains practically uniform at 427 °C, and there is no indication of thermal runaway. It represents the condition of classic Joule’s law, which assumes uniform sample and no depletion layer.

## Discussion

The recent discovery of EFIS of glass^19–21^, the present observations of localized heating and thermal runaway (Figs 2–5), and the FEA modeling (Figs 5–8) clearly demonstrate that classic macro-scale Joule’s law for homogeneous samples does not apply to the electrical heating of common glasses, indeed any ionically conducting solid, when usual metal or graphite electrodes are employed. The macro-scale asymmetry of temperature evolution has been reported also during flash sintering of oxygen anion conducting yttria-stabilized zirconia ceramics^31,32^. To understand the source of this macro-scale discrepancy, we note the case of simple resistive heating that is commonly used for melting and fining of glass melts^33,34^. This technique depends on glass resistivity and its temperature dependence as an ionic conductor in the molten phase^35^.

In the rigid glass phase, at the macro-scale, Joule’s law does not apply because the homogenous glass begins to change once an external voltage is applied across it. At the start of voltage application the homogenous glass obeys Joule’s law^26^. However, mobile ions in the glass begin to migrate toward oppositely charged electrodes to form an alkali ion depletion layer in the glass nearest the anode^20^. The resulting thin layer has much higher resistivity compared to the bulk glass, so that within one minute almost the entire poling voltage drop occurs across this layer under thermal poling conditions^20,30^. The sharp drop in voltage across the sample has been calculated and is visible in Fig. 6a for a 100 nm depletion layer on the anode side of the model with 200 V applied. At this thickness, the internal electric field reaches ~1.9 × 10^7^ V/cm whereas the externally applied field would be 200 V/cm, making the external macro-scale field negligible. The dielectric strength of pure silica is 10^7^ V/cm and therefore, the internal electric field is strong enough to change the potential energy barrier for electronic conduction within an insulating material^36,37^. At this point, dielectric breakdown may occur increasing electronic conductivity to the point where electrical energy dissipation heats the glass to a thermal runaway situation. The heat generation during runaway then could sustain the breakdown event. An alternative perspective is that heat generation from highly inhomogeneous Joule heating causes thermal runaway, which then leads to dielectric breakdown. The present results may not fully resolve this dilemma of cause and effect between dielectric breakdown and thermal runaway.

A simple approximation of power dissipation can be estimated using the voltage across the sample with the current due to ionic conductivity, which obeys Ohm’s law, as follows:

1 $$Q={\int }_{{t}_{s}}^{{t}_{f}}v\ast i\,dt\,$$

where *Q* is the thermal energy due to power dissipation from electrical losses [J], *v* is the voltage across the sample [V], *i* is the current [A], *t*_*s*_ and *t*_*f*_ are the start and finish times of EFIS [s]. This relation assumes complete conversion of electrical energy into thermal energy without loss. The thermal energy estimated from Equation 1 can then be used to calculate a corresponding temperature rise within the sample, based on simple heat transfer, with the following relation:

2 $$Q=m\ast {C}_{p}\ast {\Delta }T\,$$

where *m* is the mass of the sample [g], *C*_*p*_ is the specific heat capacity of the glass [J/g.K], and *ΔT* is the change in temperature from initial to final value [K]. This calculation assumes that heat generation is much greater than heat loss and that sample temperature is uniform throughout with constant heat capacity.

**Figure 9:**
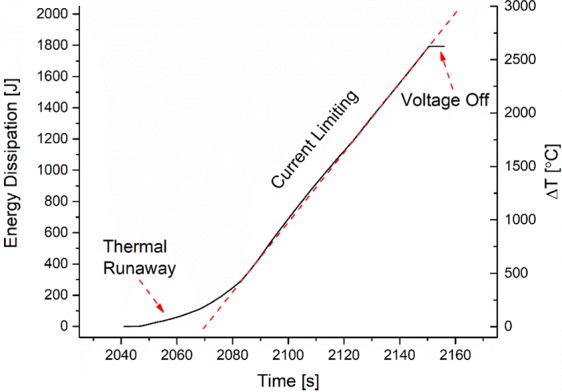
Energy dissipation and corresponding increase in sample temperature (in the limit of no heat dissipation) beyond T_furnace_ for NS glass at 150 V DC at 350 °C. Energy dissipation calculated from Eq. 1 and temperature increase from Eq. 2. Note: The red dashed line indicates the linear current limiting regime due to an in-series power resistor.

Evidently, very high temperatures can be realized within an alkali ion depletion layer next to the anode upon the simultaneous application of DC voltage and furnace heating—high enough to cause vaporization and re-deposition of glassy powder, as seen in Fig. 1a^30,38^. For example, consider NS glass with 150 V DC applied at T_furnace_ in the range of 350 °C. The energy dissipated within the glass during such a treatment was calculated from the power density using Equation 1. It is plotted in Fig. 9 for the time frame during dielectric breakdown along with the corresponding increase in sample temperature beyond T_furnace_. The change in sample temperature was approximated from Equation 2 with a specific heat capacity of 1.15 J/g.K and a mass of 0.5921 g^39^. There are two important features observed in Fig. 9. First, the energy dissipation at the onset of dielectric breakdown increases in an exponential manner, which is labeled as ‘Thermal Runaway’. The second feature is a linear increase in energy dissipation, which results from current limiting imposed by the power resistor in series with the sample. The energy dissipation eventually levels off due to the applied voltage being removed. This simple approximation gives a bounding estimate of the sample temperature increasing by 2500 °C after two minutes of current-limited thermal runaway heating. This intense heating accounts for the softening and subsequent vaporization of the glass, leaving the alkali- and silica-rich deposits seen in Fig. 1.

The plots in Fig. 9 are based on simple approximations, yet they seem to provide correctly the magnitude of temperature where glass softening and vaporization can occur. Most samples softened following ~ 30 s of thermal runaway and according to Fig. 9 the uniform temperature of the glass would be on the order of 1500 °C. To assess the validity of this assumption, thermal imaging was employed to measure actual sample surface temperature directly. It also allowed for a detailed observation of the thermal runaway process, including how the corresponding large heat input is distributed within the sample. The role of the depletion layer near the anode is clearly revealed by the temperature profile plot in Fig. 3. Here, within the first 20 s of dielectric breakdown brought about by the large current passage, very localized regions of the glass near the anode are heated up. It is believed that the localized heating corresponds to the process of thermal dielectric breakdown, which likely initiates at surface asperities or dielectric inhomogeneities wherein electric field strengths are highest locally^11^. This would explain the non-uniform heating observed in both Figs 2 and 3.

The differences in EFIS as observed between DC and AC applied voltages are believed to be due to the difference in power dissipation during EFIS for the two cases, which leads to self-heating and thermal runaway^21^. A comparison is made for NS with 150 V DC in Fig. 4 and 150 V AC at 1 kHz in Fig. 5. Note that the sample in the IR images is located in the center with the anode at the top and cathode at the bottom for the case of DC. Figure 4 compares the same NS sample with 150 V DC at two values of T_furnace_, 353.5 °C and 363.6 °C. Since the furnace heating rate was 10 °C/min and the two images were captured within 40 s of each other, the massive difference in sample temperature is attributed to thermal runaway. For the DC case in Fig. 4, the hottest temperature measurement was on the anode side of the glass similar to Fig. 2. Within 40 seconds, the sample temperature jumped by about 1,400 °C and is highly non-uniform. In the case of AC in Fig. 5, the two images were taken about 270 s apart at T_furnace_ = 385.3 °C and 428.6 °C. Between the two images, the sample temperature increased from 500.8 °C to 514.4 °C revealing that the change in T_furnace_ was actually greater than that in the glass sample, most likely due to a more uniform internal field and corresponding heat distribution under AC than in the analogous DC case. Evidently, resistive heating in AC-EFIS could be more controllable compared to the dramatic thermal runaway of DC-EFIS.

The observed difference in the IR images for DC and AC fields is supported by measured power density per unit volume. Power in DC-EFIS was calculated by voltage and current while in AC-EFIS it was calculated by RMS voltage and current [The power calculated from the RMS values of voltage and current includes a non-heating component, resulting in a power factor of less than one. Its value is difficult to determine directly due to the continuously varying temperature and depletion layer resistance, but this factor would not change the present conclusion.]. For example, NS glass tested with 150 V in DC measured a maximum power density of 91 mW/mm^3^, whereas with 150 V - 1 kHz AC it had a maximum of 55 mW/mm^3^. A similar trend was measured with 5L5NS composition with 150 V applied. In DC, it had a maximum power density of 78 mW/mm^3^, but with 150 V - 1 kHz AC the power density was 54 mW/mm^3^. It should be noted that in DC, the power dissipation is much more local near the anode compared to the AC case, which would exaggerate the difference in power density per unit volume.

The self-heating of NS in AC is uniform, and the hottest measurement was in the center of the sample between the electrodes. This profile indicates that resistive Joule heating is likely a result of the oscillating voltage that makes current flow and Joule heating start and stop every half-cycle of the frequency. By comparison, the use of DC voltage leads to extreme localized heating and glass softening, while AC contributes to uniform heating and gradual softening. As discussed in previous AC-EFIS work^21^, the application of an AC voltage creates two processes that the mobile cations undergo. In the first half-cycle of the AC voltage, the electrode has a temporary positive bias that drives mobile cations away from the electrode/glass interface into the bulk. Ion migration dominates diffusion in this half-cycle. In the second half-cycle, the bias is temporarily reversed, now there is a large cation concentration gradient driving diffusion of cations back toward the depletion layer and the reversed voltage bias drives cation migration back as well. This process likely prevents dielectric breakdown and intense localized heating as measured in DC-EFIS.

The exponential rise of current in Fig. 7a reveals the positive feedback of resistive heating. However, the rise in current reaches an asymptote showing a competition between heat generation via resistive heating, versus heat loss through convection into the electrodes and radiation into the surrounding furnace. The calculated temperatures along with the thermal profile match well with experimental measurements. Thermal imaging showed that temperatures above 1300 °C (see Fig. 2) were often reached when using DC voltages. A maximum temperature of 1868 °C was measured in NS near the depletion layer region during DC-EFIS with 150 V after ~30 s of dielectric breakdown, as seen in Fig. 4. These calculations suggest that glass softening occurs from anode toward cathode as heat transfers from depletion layer into the bulk of the glass sample. This supposition is supported by Fig. 4b. Finite element modeling predicts an overall depletion layer temperature of about 1600 °C, while infrared imaging measured about 1800 °C. The discrepancy in values could result from FEA modeling the sample as a one-dimensional solid, which does not take into account heat buildup in the center of the glass with a radial temperature gradient. The model also used temperature-independent thermal conductivity for calculations.

In the FEA model setup, a current limit using a logic statement was imposed at 0.3A, which was larger than the asymptote reached during FEA. A similar current maximum of about 0.23A was similarly measured experimentally during EFIS, as seen in Fig. 7a. The series power resistor limited the maximum current to 0.5A in theory, but was never fully reached experimentally^20^. A comparison of the theoretical and experimental current asymptotes shows that the EFIS process is self-sustaining. Current flow through the glass dissipates as heat that increases the temperature and enhances ionic migration and diffusion. However, the creation of a silica-rich region increases sample resistance, preventing further exponential increase in current. This limitation of heat generation was not observed during AC-EFIS^21^. The power dissipation continually increased during AC-EFIS but the softening event occurred at lower furnace temperature. The latter fact was reported as a result of the sample being heated more uniformly and softened gradually, as opposed to DC-EFIS which was sudden and dramatic^21^. In principle, the asymptotic current would be observed also in AC-EFIS if a low enough frequency is used to allow enough time for an alkali ion depletion layer to form and remain stable at both electrodes.

For the optimization of heat generation, the FEA simulation of temperature profiles for varying δ reveals an insightful trend in Fig. 8. For very small δ, say at 5 nm, the resistivity of the depletion layer did not create localized heating near the anode. Instead, uniform Joule heating was experienced in the bulk of the glass. The heat that was generated within the 5 nm depletion layer could dissipate quickly out into the electrode by conduction. The maximum temperature experienced by the depletion layer was when its thickness was 100 nm. A δ beyond 100 nm began to limit the amount of current that could pass through the sample due to the larger resistance of the depletion layer. In turn, the reduced current decreased the attendant Joule heating effects, as seen in Fig. 8. Consequently, thermal runaway is either extended to a longer time scale or suppressed to where heat loss is no longer negligible compared to heat generation and comes to a steady state. This insight gained by thermal modeling explains dynamic Joule heating as observed through experimental thermal imaging.

Thermal imaging videos show that the heating during EFIS near the anode is highly localized and non-uniform. The localized ‘hot spot’ also tends to meander around, laterally, at the anode/glass interface. The FEA model results explain this observation, where intense localized heating creates a large δ due to thermally-enhanced migration of cations. The δ can then grow to be on the order of 50 µm as measured by EDS linescans^21^. Upon reaching a relatively ‘thick’ depletion layer of 50 µm, the thermal runaway becomes suppressed in that local region by limited current through the more resistive layer. This process serves as a negative feedback loop of the dielectric material’s ability to sustain current in that localized area. However, in a region adjacent to the localized heating, conditions may be conducive for dielectric breakdown to continue by traversing laterally over to an adjacent region that was heated by the nearby thermal runaway event but retained an ‘optimal’ value of δ at ~100 nm. The thermal runaway process continues in this new region, until in turn it also becomes suppressed by a growing depletion layer, causing it to move to a neighboring region again, and so on. As neighboring ‘hot spot’ regions join each other, the collective resistance of the extended depletion layer will decrease, and sufficient heat will be generated to initiate EFIS. Thus, heat transfer from the depletion layer into the bulk will eventually result in the bulk sample temperature reaching softening temperatures, allowing for viscous flow to occur.

## Conclusions

The key message of this work is that electrical heating in ionically conducting glasses is substantially different from that predicted by classical Joule’s law and observed in electronically conducting metals and semiconducting materials. The difference arises due to the development of an ion depletion layer in the former, which makes the sample electrically inhomogeneous with time. The resistance of this very thin layer (≤100 nm) can be orders of magnitude higher than the underlying bulk glass. These characteristics produce extremely high local fields that can increase the temperature locally by 1400 °C or higher than the remaining glass, causing dielectric breakdown, melting, and even evaporation of the sample. Furthermore, photoemissions and localized heating are observed to occur simultaneously in the same area of glass, which meander around randomly along the anode/glass interface under the influence of a DC field. These observations call for a micro-scale version of Joule’s law that incorporates field-induced, localized, dynamic electrical inhomogeneity, and enhanced cation mobility due to localized heating.

The *in situ* measurements of sample temperature by infrared imaging, coupled with modeling of electrical heating of ionically conducting silicate glasses, provide new insights into the recently discovered EFIS phenomenon. The results show the existence of an optimum thickness of ion depletion layer for which dielectric breakdown and EFIS are most likely to occur. FEA calculations of DC-EFIS for alkali disilicate glass predict regions within the sample to reach temperatures of about 1600 °C—far above the ambient furnace temperature of 427 °C. Thermal imaging of DC-EFIS experimentally corroborates a sample surface temperature of about 1800 °C. Thermal imaging of AC-EFIS shows relatively homogeneous resistive heating, which leads to more uniform heating and softening of the entire sample. By comparison, under DC field the sample softens, starting at the anode and then moving toward cathode.

## Experimental Methods

Similar to prior studies, in these experiments we focus on a series of alkali disilicate glasses. The glasses were synthesized by mixing appropriate amounts of sodium carbonate (Na_2_CO_3_), lithium carbonate (Li_2_CO_3_) and silica (SiO_2_) powders in proportion to the nominal compositions of NS, 2L8NS and 5L5NS disilicate glasses listed in Table 1. The samples were made using the melt-quench method, starting with the mixed powders that were melted at 1550 °C for 2 h at a heating rate of 10 °C/min^19^. The cast samples were annealed for 3 h at ~35 °C below their corresponding glass transition temperature (T_g_) as measured by differential scanning calorimetry (model NETZSCH 404/F3) at 10 °C/min. The T_g_ value for each composition is listed in Table 1. Samples were ground and polished to create rectangular bars with a final cross-section of 5 mm x 5 mm and a height of 10 mm. Conductive carbon paste was applied to the end-faces of the glass samples to ensure good electrical contact with the solid graphite electrodes.

For EFIS measurements with *in situ* thermal imaging, a C-shaped bracket was used to hold graphite electrodes in contact with the sample during heating and provide a ‘backdrop’ for the camera. Either AC (60 Hz or 1 kHz) or DC voltage, ranging from 100 to 200 V, was applied across the sample, with a power resistor connected in series that limited the current to 0.5A, as previously reported^19,21^. All the bar samples were held vertically in the furnace and heated at a constant rate of 10 °C/min under a 1 MPa compressive load until DC- or AC-EFIS occurred. In DC-EFIS experiments, the positively-biased electrode (anode) was always located at the top of the image, while the cathode was kept at the bottom.

In some instances, a powdery white material was deposited on the surfaces of adjacent support structures. Samples of this powder were analyzed in a Zeiss 1550 scanning electron microscope with energy dispersive x-ray spectroscopy (EDS).

For high quality infrared (IR) imaging, the longer side faces of the bar samples were ground to 130 µm surface roughness, since a smoother surface produced reflection that altered the IR measurements. Two IR cameras were used for thermal imaging: (a) An FLIR SC8303HD-HS F/4 BHP 3–5um 1344X784 comprising a 100 mm lens with a 65 mm extension, which allowed for focusing on the sample surface 24.4 cm away, inside the furnace through an IR-transparent window. The lens of the camera looking into a furnace port was cooled with a fan that resulted in an artifact vibration in the IR videos. The camera sensor had a 1344 pixel by 784 pixel resolution, providing an ultimate resolution of 0.03 mm on the sample. Then a 3 × 3 pixel matrix was averaged, achieving a resolution of 0.1 mm. The maximum temperature reading of the FLIR camera temperature range setting was 1300 °C ± 2%. The detection wavelength range for thermal imaging was 3 µm to 5 µm. The emissivity values for the NS and 5L5NS glass compositions were measured as 0.95 and 0.96, respectively, at a wavelength of 5.14 µm ranging in temperature from 235 °C to 540 °C (see Supplementary Table 1). Thermal images were analyzed using the ResearchIR (FLIR^®^ Systems) software. (b) A Fluke TiX660 camera with the same setup as described above. However, this IR camera had a larger temperature range from −40 °C to 2000 °C ± 1.5% with a resolution on target of 0.2 mm. All the videos were recorded at 30 frames/s.

## Numerical Modeling

Numerical models of EFIS were developed using finite element analysis (FEA) in Comsol version 5.2.0.220 software. For thermal modeling of Joule heating, the electric current AC/DC module was combined with the module on heat transfer in solids using a multiphysics coupling of electromagnetic heat source and temperature. Two domains were created in series representing, respectively, the alkali ion depletion layer nearest the anode and the bulk glass properties. There are three limitations of the present FEA modeling of thermal runaway condition. First, the two domains modeling the alkali ion depletion layer and bulk of the sample are one-dimensional. Therefore, a radial temperature gradient from center-to-surface of the modeled glass is not considered. The only temperature gradient allowed is the thermal profile from anode to cathode. Second, the thermal conductivity of both the alkali ion depletion layer and bulk domains were set equal as well as temperature independent. Third, the model does not consider any heating due to current during dielectric breakdown beyond ionic conductivity under polarizing conditions.

A set of material properties was assigned to each domain. The domain representing the alkali ion depletion layer was modeled as silica glass. Its temperature dependent electrical conductivity was taken from literature values following the relation σ_Silica_ = 350*exp(−1.3 eV/k_b_*T) [S/m] where k_b_ is Boltzmann’s constant^40^. The second domain was represented by the bulk NS glass material properties. The temperature dependent ionic conductivity of bulk NS was taken from impedance spectroscopy measurements^26^. Comsol multiphysics’ nearest function interpolation method was used to extrapolate conductivities at higher sample temperatures using the following relation σ_NS_ = 12500*exp(−0.61 eV/k_b_*T) [S/m]. The change in temperature dependence from below to above T_g_ was not accounted for in this model. The thermal conductivity of both domains was set to 1.38 W/m.K, with a heat transfer coefficient for convective heat flow of 800 W/m^2^.K^41^. The heat transfer coefficient used to calculate heat convection was assumed to remain constant. The emissivity was set at the measured values of 0.95 for NS and 0.96 for 5L5NS, respectively.

The two domains consisted of three boundaries: the first outer boundary on the left of the depletion layer; the interface between depletion layer and bulk; and finally, the other outer boundary of the bulk. The first boundary (depletion layer domain) was designated as the anode with an initial voltage of 200 V applied using a terminal boundary. The right outer boundary (bulk domain) was set as the cathode, where ground potential was established. The terminal boundary was used instead of an electric potential boundary to simulate the effect of a power resistor in series with the glass sample acting as a current limiting resistor to 0.3A, if needed. Convective heat flux was allowed at the outer boundaries while a diffuse surface boundary was used for heat loss through radiation along the length of the model. A cross-sectional area of 25 mm^2^ was used to match the one used in EFIS experiments. The initial T_furnace_ for calculating thermal runaway ranged from 500 K (227 °C) to 700 K (427 °C) in 50 K intervals. Lastly, the depletion layer thickness dependence was calculated at 700 K with its value ranging from 5 nm to 50 µm.

The mesh for the depletion layer domain contained 200 elements. Typically, the δ = 100 nm would have a mesh density of 2 elements/nm. In the bulk domain, only 500 elements were used to reduce computing time. The bulk mesh density was close to 50 elements/mm. Most experiments during EFIS exhibited viscous flow of the sample within 30 s of entering region IV of EFIS associated with dielectric breakdown and thermal runaway^20^. Therefore, all thermal runaway simulations were tested from 0 to 30 s with 1 s intervals.

The current density within the two domains was calculated between each element node using Ohm’s law. The amount of energy dissipation from electrically resistive losses was then calculated following Joule’s law using current density multiplied by the local electric field strength at each element node along the one-dimensional model to give energy density, *Q*_*e*_. Assuming complete conversion of Joule energy dissipation into thermal energy, the increase in sample temperature or the temperature gradient between element nodes was calculated by relating the energy density to the previously mentioned thermal conductivity as:

3 $${Q}_{e}=\rho {C}_{P}\frac{\delta T}{\delta t}-{\nabla }(K\ast {\nabla }T)$$

where ρ is density [g/cm^3^], *C*_*P*_ is the specific heat capacity at constant pressure [J/g.K], *K* is thermal conductivity [W/cm.K] and *∇T* is the temperature gradient [K/cm] along the longitudinal profile of the sample model.

## Supplementary information

Supplement to Development of highly inhomogeneous temperature profile within electrically heated alkali silicate glasses

DC EFIS 5L5NS 200V

## Data Availability

All relevant data that supports our experimental findings are available from the corresponding author upon reasonable request.
