# Supplementary material for: Development of highly inhomogeneous temperature profile within electrically heated alkali silicate glasses
Source: Sci Rep. 2019 Feb 26;9:2805. doi: 10.1038/s41598-019-39431-8 (PMC6391379; doi:10.1038/s41598-019-39431-8)
Supplement: Supplementary file 1 — Supplement to Development of highly inhomogeneous temperature profile within electrically heated alkali silicate glasses [file 41598_2019_39431_MOESM1_ESM.docx]

**Development of highly inhomogeneous temperature profile within electrically heated alkali silicate glasses**

Charles T. McLaren^1,^^[[1]](#footnote-1)^ Craig Kopatz^2^, Nicholas J. Smith^2^, Himanshu Jain^1,^^[[2]](#footnote-2)^

^1^Department of Materials Science and Engineering, Lehigh University, Bethlehem, PA 18015, USA

^2^Corning Incorporated, Corning, NY 14830, USA

Supplementary Table 1: Measured surface emissivity of NS and 5L5NS glass compositions using the FLIR SC8303HD-HS F/4 BHP 3-5um 1344X784 infrared camera at a wavelength of 5.14 µm.

| **Temperature** | **235°C** | **440°C** | **445°C** | **450°C** | **500°C** | **540°C** |
| --- | --- | --- | --- | --- | --- | --- |
| **NS** | 0.95 |  | 0.95 |  | 0.95 | 0.95 |
| **5L5NS** |  | 0.96 |  | 0.96 | 0.96 | 0.95 |





Supplementary Figure 1: Experimental data of 5L5NS undergoing EFIS with 200 V applied and 10°C/min heating rate. The time has been adjusted to focus on just the EFIS event occurring at a furnace temperature of ~325°C.


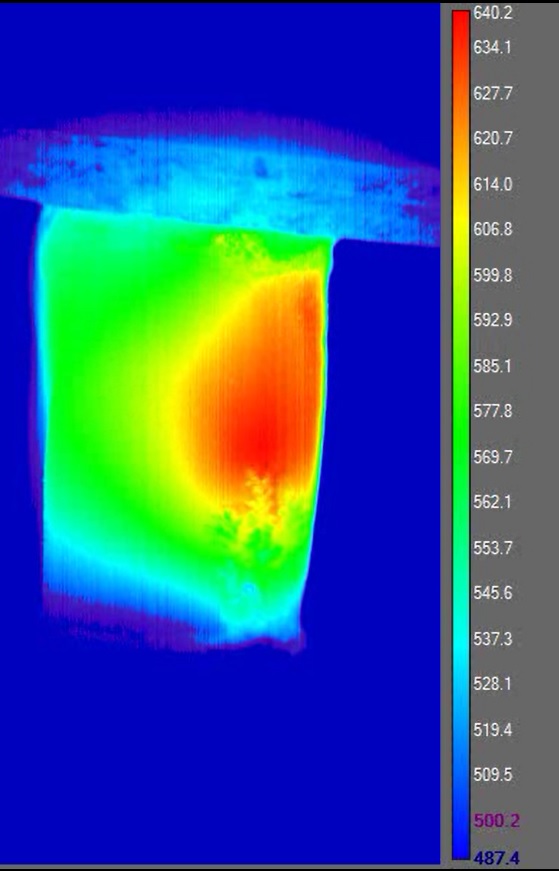

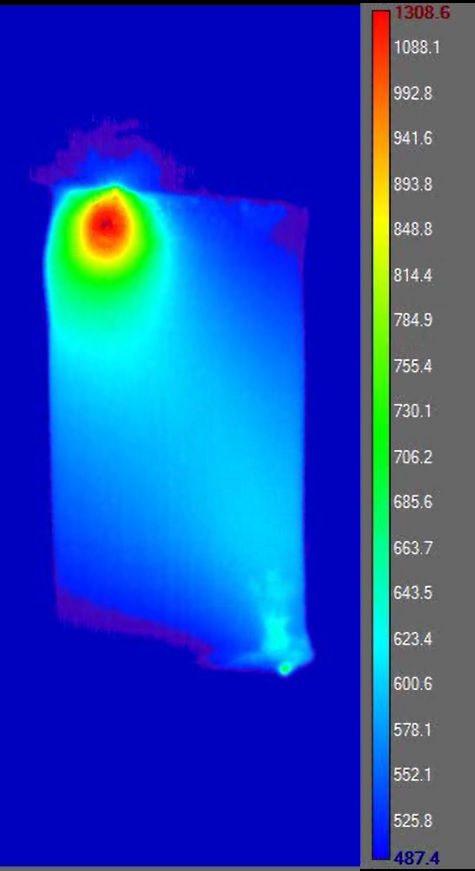


a)

b)

Supplementary Figure 2: IR images of 2L8NS with 150 V applied at 430°C furnace temperature. The formation of an alkali-rich phase can be seen growing from cathode toward anode at a) sample temperature of 575°C, and b) continued growth at 600°C. The alkali-rich phase is circled. Note: The sample has a width of 5 mm.

1. Now with Science & Technology Division, Corning Incorporated, Corning, NY 14830, USA [↑](#footnote-ref-1)
2. Corresponding author. Email: H.Jain@Lehigh.edu [↑](#footnote-ref-2)
